# Faint trace of a particle in a noisy Vaidman three-path interferometer

**DOI:** 10.1038/s41598-020-80806-z

**Published:** 2021-01-13

**Authors:** Jerzy Dajka

**Affiliations:** 1 Institute of Computer Science, University of Silesia in Katowice, 40-007 Katowice, Poland; 2 Institute of Physics, University of Silesia in Katowice, 40-007 Katowice, Poland; 3 Silesian Center for Education and Interdisciplinary Research, University of Silesia in Katowice, 41-500 Chorzów, Poland

**Keywords:** Quantum information, Qubits

## Abstract

We study weak traces of particle passing Vaidman’s nested Mach–Zehnder interferometer. We investigate an effect of decoherence caused by an environment coupled to internal degree of freedom (a spin) of a travelling particle. We consider two models: pure decoherence leading to exact results and weak coupling Davies approximation allowing to include dissipative effects. We show that potentially anomalous discontinuity of particle paths survives an effect of decoherence unless it affects internal part of the nested interferometer.

## Introduction

Quantum particle can be prepared (preselected) in a given and desired state and may also be postselected in another state with a known at least in principle probability. That what occurs in between, what is the particle’s past, remains problematic due to specific features of quantum measurements unavoidably modifying quantum states of measured objects. It is clearly visible for a quantum particle passing trough interferometer: the particle enters the device and leave it (if its outcome is measured), however, what occurs inside an interferometer is, as it will be shown below, disputable and even controversial. One of the approaches^1,2^ is that a particle, staying coherent, it leaves nothing but a faint trace which—if comparable to an order of a trace potentially left by a localized wave packet—can serve as a hallmark of its presence. A faint trace which is a *small* change of an amplitude of a component orthogonal to an undisturbed particle’s state (weakly) measurable only in experiments with an ensemble of particles having the same pre-and postselection.

**Figure 1:**
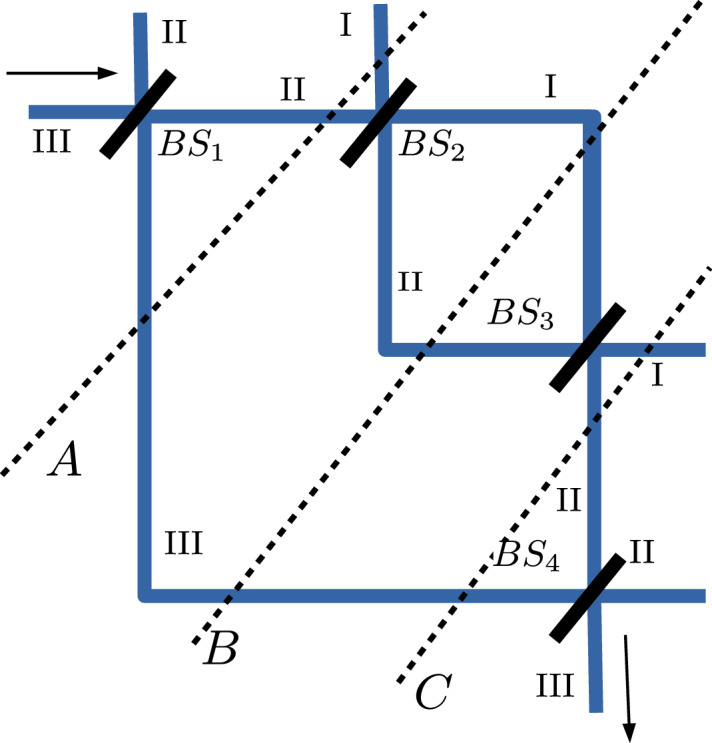
Vaidman interferometer consisting of two Mach–Zehnder nested devices with four beam splitters $BS_i, i=1,\ldots ,4$. Roman numbers $\hbox {I},\hbox {II},\hbox {III}$ correspond to vectors in Eq. (1) and *A*, *B*, *C* denote instants where the weak trace is calculated.

Past of a quantum particle in a nested Mach–Zehnder interferometer—proposed in Ref.^1^ and presented in Fig. 1 and abbreviated here as a Vaidman interferometer—was recently studied in Ref.^1^ using quantum weak values^3,4^ and the two state vector formalism (TSVF)^5^. The results are confounding: particles seem to follow anomalous *discontinuous* path. Such a seemingly weird conclusion leads to plethora of controversies^6,7^ and since that time (almost) all works on that problem have came in triads: a paper, commentary inspired by the paper and a reply to the comment^6,7^. The main reason is that the TSVF^5^ applied in Ref.^1^ is one of few possible approaches to studies of quantum past. The other non-equivalent alternatives are consistent (decoherent) histories^8,9^ standard quantum mechanics^10–12^ and many other other alternative studies^13–20^. Moreover, even recent experiments and their detailed analyses fail to resolve all the controversial issues^20–26,26–28^. Despite counter-intuitive of a conception of discontinuous path there are analyses^2,29^ and claims which support the faint-trace anomalous picture as experimentally confirmed.

Our present aim is to follow Ref.^1,2^ and to supplement the analysis of weak trace (based upon weak values) of particle by including an effect of decoherence affecting internal degree of freedom of the particle passing the Vaidman interferometer. Our results allow to identify natural obstructions for an effective verification of theoretical predictions and exclude factors seemingly but not truly responsible for experimental failures and limitations. Studying internal degrees of freedom e.g. spin or polarization of particles in Vaidman interferometer becomes particularly reasonable for most recent experiments and models utilizing neutrons^24,26^. There are various phenomenologic approaches^30^ dedicated to particular quantum systems which usefulness and validity was confirmed by many repeatable experiments. However, for fragile quantum systems microscopic models are a least a good starting point to make predictions which are credible in a tailored and well identified conditions. In this paper we consider two well established microscopic models: *(i)* an exact model of pure decoherence^31,32^ of a solely quantum character and *(ii)* a weak coupling Davies approximation^33^ allowing to include dissipation. Pure decoherence (or pure dephasing) model is limited by a choice of system–environment interaction encoded in a Hamiltonian by an integral of motion: in the Caldeira–Leggett^30^ “system + bath + interaction” Hamiltonian the interaction is given by an operator commuting with a system. Davies approach^33^ allows for an arbitrary system–environment coupling provided that its small enough for a perturbative treatment to hold true. We show that that the anomalous discontinuity^1,2^ of the faint traces (given by weak values of suitable projection operators) left by particles are rigid with respect to decoherence affecting external arm of Vaidman interferometer while very fragile if a source of decoherence disturbs balance of the internal, nested arms of Vaidman interferometer. Since an experiment is an only way to resolve an (interpretative) ambiguity concerning (un)presence of particles in the Vaidman interferometer the answer if the controversies survive also in the presence of decoherence seems to be crucial.

For a sake of completeness we re-introduce the Vaidman interferometer and review the controversial features of the faint traces of particles passing it. The original Vaidman interferometer presented in Fig. 1 consists of spatial degree of freedom given by three paths denoted by $\hbox {I},\hbox {II},\hbox {III}$ and four beam splitters. Vaidman interferometer can effectively be described^10–12^ as a three level quantum system with a state space spanned by a basis

1 $$\begin{aligned} |\hbox {I}\rangle =\left( \begin{array}{c} 1 \\ 0 \\ 0 \end{array}\right) ,\,\,|\hbox {II}\rangle =\left( \begin{array}{c} 0 \\ 1 \\ 0 \end{array}\right) \text{ and }\,\,\,\, |\hbox {III}\rangle =\left( \begin{array}{c} 0 \\ 0 \\ 1 \end{array}\right) \end{aligned}$$

In an ideal setting of a noise-less system, a passage of a particle is described by a unitary transformation composed of four unitaries $U_4U_3U_2U_1$ corresponding to subsequent beam splitters termed as $BS_{i}, i=1, \ldots , 4$ in Fig. 1:

2 $$\begin{aligned} U_1 = U_4 =\frac{1}{\sqrt{3}}\left( \begin{array}{ccc} \sqrt{3} &{} 0 &{} 0 \\ 0 &{} -1 &{} \sqrt{2} \\ 0 &{} \sqrt{2} &{} 1 \end{array}\right) \,\, \text{ and }\,\,\,\, U_2 = U_3 =\frac{1}{\sqrt{2}}\left( \begin{array}{ccc} 1 &{} 1 &{} 0 \\ -1 &{} 1 &{} 0 \\ 0 &{} 0 &{} \sqrt{2} \end{array}\right) \end{aligned}$$

The strategy applied in Ref.^1^ to infer the path of a particle entering and leaving Vaidman interferometer in a state $|\hbox {III}\rangle$ was to investigate *weak trace* of a particle inside Vaidman interferometer at three instants *A*, *B*, *C* indicated in Fig. 1. According to Ref.^1^ the weak trace is indicated by a non-vanishing weak value^3,34^ of one of the projectors

3 $$\begin{aligned} \langle \Pi _i\rangle _w^q= & {} \frac{\langle \psi _{post}^q|\Pi _i|\psi _{pre}^q\rangle }{\langle \psi _{post}^q|\psi _{pre}^q\rangle }, \,\,\, \text{ where }\,\,\,\,\Pi _i = |i\rangle \langle i|,\,\, i=\hbox {I},\hbox {II},\hbox {III} \,\,\, \text{ and } \,\,\, q=A,B,C \end{aligned}$$

where preselected (directly prior to the measurement of $\Pi _i$) and postselected (immediately after the measurement) states compose a *two-state vector* $\langle \psi _{post}||\psi _{pre}\rangle$ being a fundamental object of the TSVF^5^.

Let us emphasise that the physical meaning of vanishing weak values in a current context remains disputable^34–37^. Most of controversies originate, however, from highly counter-intuitive conclusions provided in Ref.^1^ indicating possibility of discontinuous trajectories followed by a particle passing trough Vaidman interferometer. Let us review: there are three instants *A*, *B*, *C* where the weak trace is measured: *A*: just after it is injected to the Vaidman interferometer in a state $|\hbox {III}\rangle$ and passes $BS_1$, *B*: where the weak measurement becomes conducted for all potential paths in Vaidman interferometer and *C*: after the $BS_3$ beam splitter as presented in Fig. 1. The corresponding preselected states read as: $|\psi _{pre}^A\rangle = U_1|\hbox {III}\rangle$, $|\psi _{pre}^B\rangle = U_2U_1|\hbox {III}\rangle$ and $|\psi _{pre}^C\rangle = U_3U_2U_1|\hbox {III}\rangle$. In the time-symmetric TSVF setting the postselected states $|\psi _{post}^A\rangle = U^\dagger _2U^\dagger _3U^\dagger _4|\hbox {III}\rangle$, $|\psi _{post}^B\rangle =U^\dagger _3U^\dagger _4|\hbox {III}\rangle$ and $|\psi _{post}^C\rangle =U^\dagger _4|\hbox {III}\rangle$ describe a hypothetical particle detected at $\hbox {III}$ evolving backward in time. Results of the weak measurement are summarized in Table 1. According to Ref.^1^ a presence of a particle is defined by its non-vanishing weak trace. The counter-intuitive conclusion of Table 1 is the following: at *A* and *C* the particle is present in $\hbox {III}$, what upon Fig. 1 is intuitively acceptable, but at *B* it is also present in an internal loop ($\hbox {I},\hbox {II}$) of Vaidman interferometer. Such confounding result needs experimental verification. One can safely assume that any potential experiment, as it was so far, will be highly subtle and sophisticated. Moreover, such an experiment will rely on quantum properties and it may be fragile with respect to decoherence. Our objective is to investigate if in a presence of omnipresent noise one can still support claims inferred from Table 1 or if they are nothing but an artifact absent in real noisy systems.

**Table 1:** Weak traces $\langle \Pi _{\mathrm{I},\mathrm{II},\mathrm{III}}\rangle _w^{A,B,C}$ Eq. (3) of a particle in a noise-less Vaidman interferometer at different instants *A*, *B*, *C* indicated in Fig. 1 and corresponding pre-and postselections given by $|\psi _{pre}^{A,B,C}\rangle =U_{pre}^{A,B,C}|\hbox {III}\rangle$ and $|\psi _{post}^{A,B,C}\rangle =U_{post}^{A,B,C}|\hbox {III}\rangle$ respectively.

$\langle \Pi _{\mathrm{I},\mathrm{II},\mathrm{III}}\rangle _w^{A,B,C}$	$\hbox {I}$	$\hbox {II}$	$\hbox {III}$	$U_{pre}^{A,B,C}$	$U_{post}^{A,B,C}$
A	0	0	1	$U_1$	$U^\dagger _2U^\dagger _3U^\dagger _4$
B	− 1	1	1	$U_2U_1$	$U^\dagger _3U^\dagger _4$
C	0	0	1	$U_3U_2U_1$	$U^\dagger _4$

## Pure decoherence

There are circumstances when internal degrees of freedom of particles need to be taken into account and affect an interference^38^. An output pattern of such an interference becomes further significantly modified by external bath coupling to an internal degree of freedom^39^. In the following for simplicity we assume that an internal degree of freedom of a particle in the Vaidman interferometer requires two-dimensional space $\mathscr {H}_{int}$ spanned by

4 $$\begin{aligned} |+\rangle =\left( \begin{array}{c} 1 \\ 0 \end{array}\right) ,\,\,|-\rangle =\left( \begin{array}{c} 0 \\ 1 \end{array}\right) \end{aligned}$$

Physically, such a qubit model can correspond to an interference of spin-half particles or any qubits. If, moreover, one assumes the internal degree of freedom is coupled to (affected by) an environment one arrives at a composite quantum system with a state space

5 $$\begin{aligned} \mathscr {H}= & {} \mathscr {H}_{path}\otimes \mathscr {H}_{int}\otimes \mathscr {H}_{env} \end{aligned}$$

where $\mathscr {H}_{int}=\mathbf {C}^2$ and $\mathscr {H}_{path}=\mathbf {C}^3$ with a basis $|I\rangle ,|II\rangle ,|III\rangle$ in Eq. (1). As a composite system consisting of a particle and its environment considered *in toto* is closed, its evolving states undergo unitary transformations. Unitaries corresponding to beam splitters Eq. (2) and projectors Eq. (3) required for weak measurement become now tensorized with identity operators $\mathbb {I}$ acting on remaining parts of a composite space $\mathscr {H}$ and read:

6 $$\begin{aligned} \mathbf {U}_i= & {} U_i\otimes \mathbb {I}_2 \otimes \mathbb {I}_{env}:\mathscr {H}\rightarrow \mathscr {H},\,\,\, i=1, \ldots , 4 \end{aligned}$$

7 $$\begin{aligned} \tilde{\Pi }_i= & {} \Pi _i \otimes \mathbb {I}_2 \otimes \mathbb {I}_{env}:\mathscr {H}\rightarrow \mathscr {H},\,\,\, i=\hbox {I},\hbox {II}, \hbox {III} \end{aligned}$$

where $U_i$ and $\Pi _i$ are given in Eqs. (2) and (3) respectively.

Let us notice that an effect of a generic interaction between particle and its environment shall result in a modification of $\mathbf {U}_i$ which will essentially be interaction-type-dependent. Pure decoherence (dephasing)^30,31^ is probably the simplest class of an interaction between an open quantum system and its environment. It is characterized by a high symmetry preventing energy exchange with a surrounding^31^. Despite such an obvious simplification pure decoherence can effectively be applied to a broad class of problems^40–47^ ranging from theoretical quantum information up to experiments in optics. Simplifying our model even further we assume *local* decoherence i.e. the particle remains unaffected by a dephasing environment unless it follows a particular ’noisy’ path between two particular beam splitters. The term ’local’ is used to distinguish circumstances when a whole Vaidmann interferometer, not just a part of of one its arms, is embedded in an either thermal or non-thermal bath. Hamiltonian describing interaction between particle (which is a qubit with a Hamiltonian given by $\sigma _z$ Pauli matrix) and its environment is then given by a standard Caldeira–Leggett form^30^:

8 $$\begin{aligned} H_{i}= & {} E\mathbb {I}_3\otimes \sigma _z\otimes \mathbb {I}_{env} +\Pi _i\otimes \sigma _z\otimes \int _0^\infty d\omega (g(\omega )a(\omega )+h.c.)+ \mathbb {I}_3\otimes \mathbb {I}_2\otimes \int _0^\infty d\omega h(\omega )a^\dagger (\omega )a(\omega ),\,\,i=\hbox {I},\hbox {II},\hbox {III} \end{aligned}$$

Time evolution of a total (closed) system is unitary and reads

9 $$\begin{aligned} U_{i}= & {} \exp (-itH_i)=|i\rangle \langle i|\otimes \left( \begin{array}{cc} U_+ &{} 0\\ 0 &{} U_- \end{array}\right) + (\mathbb {I}_3-|i\rangle \langle i|)\otimes \mathbb {I}_2 \otimes \mathbb {I}_{env}, \,\,\, i=\hbox {I},\hbox {II}, \hbox {III} \end{aligned}$$

10 $$\begin{aligned} U_\pm= & {} \exp (-itH_\pm ): \mathscr {H}_{env} \rightarrow \mathscr {H}_{env} \end{aligned}$$

where *t* denotes duration of particle–bath interaction which is assumed to be smaller that the passage between any pair of beam splitters. A block–diagonal structure of the first term in Eq. (9) with unitary blocks $U_\pm$ given by Eq. (10) is a hallmark of the assumed pure decoherence model with

11 $$\begin{aligned} H_\pm= & {} \int _0^\infty d\omega \left[ h(\omega ) a^\dagger (\omega )a(\omega ) \pm (g(\omega )a(\omega )+h.c)\right] \pm E \end{aligned}$$

where *E* denotes energy separating qubit levels, the environment is simplified to a one-dimensional bosonic field with bosonic operators $a(\omega ),a^\dagger (\omega )$ and real-valued $h(\omega )$ and $g(\omega )$. To clarify the notation of Eq. (9) let us exemplify: a unitary transformation $\mathbf {U}_4\mathbf {U}_3\mathbf {U}_2U_{\mathrm{II}}\mathbf {U}_1:\mathscr {H}\rightarrow \mathscr {H}$ describes Vaidman interferometer with a purely dephasing environment coupled to a path $\hbox {II}$ locally between beam splitters $BS_1$ and $BS_2$.

Now we are ready to analyse an impact of decoherence on particles travelling via Vaidman interferometer. We recognize two classes of effects: the first, quantitative when the anomalous effect of Ref.^1^ survives and the second when the pure decoherence spoils unusual features of noise-less system. The first case in presented in Table 2. We consider quantum particle entering Vaidman interferometer in a state

12 $$\begin{aligned} |\psi _0\rangle= & {} |\hbox {III}\rangle \otimes [|+\rangle + |-\rangle ]/\sqrt{2} \end{aligned}$$

where the first, spatial, component denotes path of the particle whereas the second is a state of internal degree of freedom (qubit). The particle leaving the Vaidman interferometer is assumed to be in a state

13 $$\begin{aligned} \rho _{out}= & {} |\hbox {III}\rangle \langle \hbox {III}|\otimes \frac{1}{2}[|+\rangle \langle +|+|-\rangle \langle -|]=|\hbox {III}\rangle \langle \hbox {III}|\otimes \frac{1}{2}\mathbb {I}_2 \end{aligned}$$

i.e. it is postselected in its spatial (external) degree of freedom but with no information regarding its internal (here maximally mixed) state. Moreover, we assume that an environment both initially and finally is its ground state (vacuum) $|\Omega \rangle$ and affects only these particles which, according to TSVF, travel forward in time. Working essentially with mixed states requires generalisation of a definition of a weak value of an operators^34^. In particular for a faint trace one calculates

14 $$\begin{aligned} \langle \Pi _{\mathrm{I},\mathrm{II},\mathrm{III}}\otimes \mathbb {I}_2\rangle _w= & {} \frac{\text{ Tr }(\rho _{post}[\Pi _{\mathrm{I},\mathrm{II},\mathrm{III}}\otimes \mathbb {I}_2]\rho _{pre})}{\text{ Tr }(\rho _{pre}\rho _{post})} \end{aligned}$$

for generically mixed pre-and postselections. Depending on the instants *A*, *B*, *C*, cf. Fig. 1 of a weak measurement, we consider three different pre-and postselection $\rho _{pre}^{A,B,C}=\mathbf {U}_{pre}^{A,B,C}(|\psi _0\rangle \langle \psi _0|\otimes |\Omega \rangle \langle \Omega |)[\mathbf {U}_{pre}^{A,B,C}]^\dagger$ and $\rho _{post}^{A,B,C}=\mathbf {U}_{post}^{A,B,C}(\rho _{out}\otimes |\Omega \rangle \langle \Omega |)[\mathbf {U}_{post}^{A,B,C}]^\dagger$ respectively with $\mathbf {U}_{pre,post}^{A,B,C}$ given explicitly in Tables 2, 3. The results of weak measurement of a particle disturbed by decoherence coupled either to $\hbox {II}$ or $\hbox {III}$ are summarized in Table 2. Let us notice that decoherence affects particle traveling forward in time after it passes the beam splitter $BS_1$.

**Table 2:** Weak traces $\langle \tilde{\Pi }_{\mathrm{I},\mathrm{II},\mathrm{III}}\rangle _w^{A,B,C}$ Eq. (7) of a particle in a noisy Vaidman interferometer at different instants *A*, *B*, *C* indicated in Fig. 1 and corresponding pre-and postselections given by $\rho _{pre}^{A,B,C}=\mathbf {U}_{pre}^{A,B,C}(|\psi _0\rangle \langle \psi _0|\otimes |\Omega \rangle \langle \Omega |)[\mathbf {U}_{pre}^{A,B,C}]^\dagger$ and $\rho _{post}^{A,B,C}=\mathbf {U}_{post}^{A,B,C}(\rho _{out}\otimes |\Omega \rangle \langle \Omega |)[\mathbf {U}_{post}^{A,B,C}]^\dagger$ respectively. The quantity $q=\langle \Omega | U_+\Omega \rangle +\langle \Omega | U_-\Omega \rangle$ is given in Eq. (15) with $U_\pm$ given in Eq. (10). The effect of pure decoherence indicated by $U_{\mathrm{II}}$ occurs for path $\hbox {II}$ (by $U_{\mathrm{III}}$ for path $\hbox {III}$) after beam splitter $BS_1$.

$\langle \tilde{\Pi }_{\mathrm{I},\mathrm{II},\mathrm{III}}\rangle _w^{A,B,C}$	$\hbox {I}$	$\hbox {II}$	$\hbox {III}$	$\mathbf {U}_{pre}^{A,B,C}$	$\mathbf {U}_{post}^{A,B,C}$	$\mathbf {U}_{pre}^{A,B,C}$	$\mathbf {U}_{post}^{A,B,C}$
A	0	0	1	$U_{\mathrm{II}}\mathbf {U}_1$	$\mathbf {U}^\dagger _2\mathbf {U}^\dagger _3\mathbf {U}^\dagger _4$	$U_{\mathrm{III}}\mathbf {U}_1$	$\mathbf {U}^\dagger _2\mathbf {U}^\dagger _3\mathbf {U}^\dagger _4$
B	-q/2	q/2	1	$\mathbf {U}_2U_{\mathrm{II}}\mathbf {U}_1$	$\mathbf {U}^\dagger _3\mathbf {U}^\dagger _4$	$\mathbf {U}_2U_{\mathrm{III}}\mathbf {U}_1$	$\mathbf {U}^\dagger _3\mathbf {U}^\dagger _4$
C	0	0	1	$\mathbf {U}_3\mathbf {U}_2U_{\mathrm{II}}\mathbf {U}_1$	$\mathbf {U}^\dagger _4$	$\mathbf {U}_3\mathbf {U}_2U_{\mathrm{III}}\mathbf {U}_1$	$\mathbf {U}^\dagger _4$

**Table 3:** Weak traces $\langle \tilde{\Pi }_{\mathrm{I},\mathrm{II},\mathrm{III}}\rangle _w^{B,C}$ Eq. (7) of a particle in a noisy Vaidman interferometer at different instants *B*, *C* indicated in Fig. 1 and corresponding pre-and postselections given by $\rho _{pre}^{B,C}=\mathbf {U}_{pre}^{B,C}(|\psi _0\rangle \langle \psi _0|\otimes |\Omega \rangle \langle \Omega |)[\mathbf {U}_{pre}^{A,B,C}]^\dagger$ and $\rho _{post}^{B,C}=\mathbf {U}_{post}^{B,C}(\rho _{out}\otimes |\Omega \rangle \langle \Omega |)[\mathbf {U}_{post}^{B,C}]^\dagger$ respectively. The quantity $q=\langle \Omega | U_+\Omega \rangle +\langle \Omega | U_-\Omega \rangle$ with $U_\pm$ given in Eq. (10). The effect of pure decoherence indicated by $U_{\mathrm{I}}$ occurs for path $\hbox {I}$ after beam splitter $BS_2$. The effect of pure decoherence indicated by $U_{\mathrm{II}}$ occurs for path $\hbox {II}$ after beam splitter $BS_2$.

$\langle \tilde{\Pi }_{\mathrm{I},\mathrm{II},\mathrm{III}}\rangle _w^{B,C}$	$\hbox {I}$	$\hbox {II}$	$\hbox {III}$	$\mathbf {U}_{pre}^{B,C}$	$\mathbf {U}_{post}^{B,C}$	$\mathbf {U}_{pre}^{B,C}$	$\mathbf {U}_{post}^{B,C}$
B	-(q+2)/4	(q+2)/4	1	$U_{\mathrm{I}}\mathbf {U}_2\mathbf {U}_1$	$\mathbf {U}^\dagger _3\mathbf {U}^\dagger _4$	$U_{\mathrm{II}}\mathbf {U}_2\mathbf {U}_1$	$\mathbf {U}^\dagger _3\mathbf {U}^\dagger _4$
C	0	(2-q)/2	q/2	$\mathbf {U}_3U_{\mathrm{I}}\mathbf {U}_2\mathbf {U}_1$	$\mathbf {U}^\dagger _4$	$\mathbf {U}_3U_{\mathrm{II}}\mathbf {U}_2\mathbf {U}_1$	$\mathbf {U}^\dagger _4$

Modifications of weak values due to decoherence reported in Tables 2 and 3 are qualified by

15 $$\begin{aligned} q= & {} \langle \Omega | U_+\Omega \rangle +\langle \Omega | U_-\Omega \rangle \end{aligned}$$

which for pure decoherence has an exact solution^30–32^. Since expressed in terms of displacement operators *D*^48^ time evolution of a vacuum $|\Omega \rangle \in \mathscr {H}_{env}$^39^ reads

16 $$\begin{aligned} U_\pm |\Omega \rangle =\exp (\mp iEt)D\left( \pm \frac{g(\omega )}{h(\omega )}(1-e^{-ih(\omega )t}) \right) |\Omega \rangle , \,\,\,\,\,\, D(f)=\exp \left[ \int _0^\infty d\omega \left( f(\omega )a^\dagger (\omega ) -h.c.\right) \right] \end{aligned}$$

for a typical choice $h(\omega )=\omega$ and $g^2(\omega )=\lambda \omega ^{1+\mu }\exp (-\omega /\omega _c)$^30^ one can calculate explicitly

17 $$\begin{aligned} q= & {} 2\cos (Et)\exp \left( -\int _0^\infty d\omega \frac{g^2(\omega )}{h^2(\omega )}(1-\cos (h(\omega t)) \right) = 2\cos (Et)\exp \left( -\lambda \Gamma (\mu )\omega _c^\mu \frac{1-\cos (\mu \arctan (\omega _c t))}{(1+\omega _c^2t^2)^{\mu /2}}\right) \end{aligned}$$

where the parameter $\lambda$ denotes strength of a particle–environment coupling and $0\le \mu$ allows to classify environment^30^ as Ohmic $\mu =0$ or super-Ohmic for $\mu >0$. The sub-Ohmic regime $\mu <0$ suffers from known^31^ mathematical difficulties and is not considered. Let us notice that for $t=0$ the integrand in Eq. (17) vanishes, $q=2$ and the results of Ref.^1^ are reproduced. It holds also true for $\lambda \rightarrow 0$ corresponding to a particle *uncoupled* with its environment. We also conclude that even in a long time limit $t\rightarrow \infty$ the quantity $q>0$ i.e. it remains finite. Therefore we infer that discontinuous faint trace changes only quantitatively. However, there is a qualitative change if decoherence is present in an *internal* interferometer of Vaidman interferometer as presented in Table 3 i.e. if an environment is coupled either to $\hbox {I}$ or $\hbox {II}$ after beam splitter $BS_2$. In both cases, still assuming that particles travelling forward in time are affected by decoherence, we observe that a faint trace of a particle contributes to $\hbox {II}$ after $BS_3$. This observation supports claim of Ref.^10^ connecting the faint trace discontinuity with a perfect balance of internal interferometer leading to destructive interference on its output. If decoherence affects internal loop in the Vaidman interferometer it removes anomaly of a faint trace. To summarize, the faint trace, otherwise fragile, remains resistant with respect to decoherence present in an external arm of Vaidman interferometer.

## Dissipation

Rigidity of faint traces with respect to decoherence affecting external interferometer of Vaidman interferometer accompanied by its fragility with respect to decoherence present in an internal loop was analysed in previous paragraph for a very special model of pure decoherence. Here we investigate if the above predictions survive also in a presence of dissipation i.e. particle–environment realistic interaction not excluding an energy exchange. Such a problem, contrary to exactly solvable pure decoherence, demands approximate treatment^30^. We apply Davies approach^33^, one of rigorous approaches to quantum open systems dedicated to weak coupling to an environment^30^. Davies treatment, mathematically rigorous, can effectively be applied in various areas of quantum information^49–52^.

Let us keep previous assumption of decoherence affecting locally an external arm of Vaidman interferometer only and consider particle–environment interaction encoded in one of two following Hamiltonians

18 $$\begin{aligned} H_{i}= & {} E\mathbb {I}_3\otimes \sigma _z\otimes \mathbb {I}_{env} +\varepsilon \Pi _i\otimes \sigma _x\otimes \int _0^\infty d\omega (g(\omega )a(\omega )+h.c.)+ \mathbb {I}_3\otimes \mathbb {I}_2\otimes \int _0^\infty d\omega h(\omega )a^\dagger (\omega )a(\omega ), i=\hbox {II},\hbox {III} \end{aligned}$$

Here $\varepsilon$ is assumed to be small. The choice of coupling (via $\sigma _x$ Pauli matrix) is complementary to the pure decoherence model studied in the previous Section and allows us to verify if previously predicted stability of faint trace holds also true disturbed by dissipation. None of two Hamiltonians in Eq. (18) allows for an exact treatment similar to pure decoherence. Instead we assume vanishing temperature limit $T=0$ and utilize Davies approximation to find time evolution of reduced (with respect to an environment) density matrix of a particle $\rho (t)\in \mathscr {B}(\mathscr {H}_{path}\otimes \mathscr {H}_{int})$ in terms of a Master equation

19 $$\begin{aligned} \frac{d}{dt}\rho (t)= & {} -i\left[ \left( E\mathbb {I}_3\otimes \sigma _z+ \sum _{k,l=1}^6s(\Omega _{kl}) A_{kl}^{(i)\dagger }A_{kl}^{(i)}\right) ,\rho (t) \right] + \frac{1}{2}\sum _{k,l=1}^6 c(\Omega _{kl})([A_{kl}^{(i)}\rho (t), A^{(i)\dagger }_{kl}]+[A_{kl},\rho (t)A^{(i)\dagger }_{kl}]) \end{aligned}$$

with $A_{kl}^{(i)}= P_k(\varepsilon \Pi _i\otimes \sigma _x)P_l$, $i=\hbox {II},\hbox {III}$, $c(x) = 2\pi x\exp (-x/\omega _c)\Theta (x)$ and numerically $2\pi s(x)=\text{ PV }\int _{-\infty }^\infty dy [c(y)/(y-x)]$. For any initial density matrix $\rho _0$ any solution of Eq. (19) is a one-parameter completely positive quantum dynamical semi-group^53^ $\rho (t)=\mathscr {D}[\rho _0]$.

**Figure 2:**
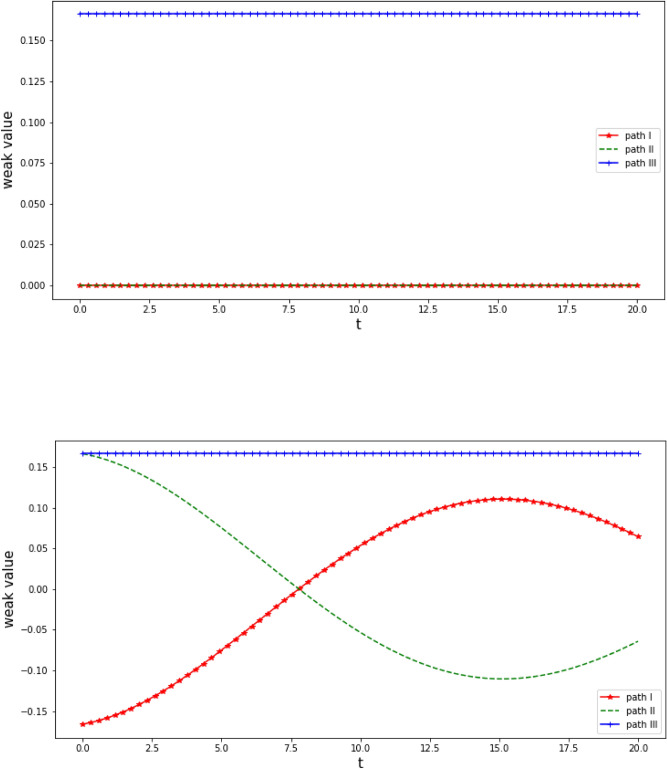
Weak values of $\langle \Pi _{\mathrm{I},\mathrm{II},\mathrm{III}}\otimes \mathbb {I}_2\rangle _w$ Eq. (14) *upper panel* at instants *A* and *C*, cf. Fig. 1, for a pre-and a postselection given in Eqs. (20) and (22) respectively and, *lower panel*, at *B* for a pre-and postselection given in Eq. (21) as a function of duration of particle-environment interaction. Time is given in $1/\omega _c$ and we set $\varepsilon =0.05$.

We investigate weak traces of a particle in Vaidman interferometer assuming that there is dissipative environment affecting particle just after $BS_1$ and it is attached to the external interferometer in the Vaidman interferometer in Fig. 1. To calculate $\langle \Pi _{\mathrm{I},\mathrm{II},\mathrm{III}}\otimes \mathbb {I}_2\rangle _w$ at instants *A*, *B*, *C*, cf. Fig. 1, for a particle injected to the Vaidman interferometer in a state $|\psi _{0}\rangle =|\hbox {III}\rangle \otimes [|+\rangle + |-\rangle ]/\sqrt{2}\in \mathscr {H}_{path}\otimes \mathscr {H}_{int}$ Eq. (12) we consider the following pre-and postselections:

20 $$\begin{aligned} \rho ^A_{pre}= & {} \mathscr {D}[U_1\otimes \mathbb {I}_2 |\psi _0\rangle \langle \psi _0| U_1^\dagger \otimes \mathbb {I}_2],\,\,\,\,\,\,\,\, \rho ^A_{post}= U_2^\dagger \otimes \mathbb {I}_2\rho ^B_{post} U_2\otimes \mathbb {I}_2 \end{aligned}$$

21 $$\begin{aligned} \rho ^B_{pre}= & {} U_2\otimes \mathbb {I}_2\rho ^A_{pre} U_2^\dagger \otimes \mathbb {I}_2 ,\,\,\,\,\,\,\,\, \rho ^B_{post}= U_3^\dagger \otimes \mathbb {I}_2\rho ^C_{post}U_3\otimes \mathbb {I}_2 \end{aligned}$$

22 $$\begin{aligned} \rho ^C_{pre}= & {} U_3\otimes \mathbb {I}_2 \rho _{pre}^B U_3^\dagger \otimes \mathbb {I}_2 ,\,\,\,\,\,\,\,\, \rho ^C_{post}= U_4^\dagger \otimes \mathbb {I}_2\rho _{out}U_4\otimes \mathbb {I}_2 \end{aligned}$$

with $\rho _{out}$ given in Eq. (13) and hence we avoid discussing decohering states evolving backward in time needed for TSVF^34^. Weak values Eq. (14) indicating faint trace of a particle in Vaidman interferometer at *A*, *B*, *C* in Fig. 1 are calculated numerically and presented in Fig. 2 as a function of duration (time) of interaction between the particle and its environment which is coupled to lower ($\hbox {III}$) arm of the external interferometer in the Vaidman interferometer in Fig. 1 after $BS_1$. Let us notice that none of the calculated weak values has a non-vanishing imaginary part requiring careful physical interpretation^4^. Weak trace calculated at *A*, *C* (*B*) is presented in the upper (lower) panel of Fig. 2 respectively. The weak trace indicating particle at *A* and *C* is present for the path $\hbox {III}$ and absent otherwise. At *B* the particle leaves weak trace in all the three paths provided that the duration (time) of dissipation is short with respect to a passage time. If it is not the case one obtains oscillations which, for certain value of duration time lead to decreasing of weak traces at $\hbox {I}$ and $\hbox {II}$ to a level which may be undetectable and, effectively but not factually, the particle remains ’visible’ in $\hbox {III}$ only. For an environment weakly disturbing path $\hbox {II}$ after $BS_1$ one obtains qualitatively similar results.

## Discussion

Decoherence is a trespasser of failure of many experiments attempting to predict or confirm quantum properties of Nature. Recent predictions of discontinuous path of particle in a nested Mach–Zehnder interferometer can serve as a particular example of a deeply quantum effect requiring further experimental verification. One could have doubt in precise measureability of the controversial and to some extent exotic properties of Ref.^1^ and in particular Ref.^2^ due to omnipresent noise blurring results of measurements. To dispel such doubts we investigated how decoherence can affect theoretical predictions of noise-less models and if it can obscure or even definitely indisposed theoretically predicted anomalies. Since recent experiments^24,26^ utilized neutrons which are particles with internal, spin degree of freedom we consider interference of qubits and decoherence affecting its spin. From a wide spectrum of different models describing quantum open systems we select two limiting cases: *(i)* an exact, non-Markov but specific pure dephasing model and *(ii)* a very general but approximate weak coupling Davies approximation. Pure decoherence, being exact, does not take into account dissipation of energy how the Davies approach does but at a cost of applied approximations. However, the results obtained for this seemingly far models were confluent: qualitative properties of weak traces (and their discontinuity) of a particle in the Vaidman interferometer are rigid with respect to decoherence affecting external interferometer but at the same time extraordinarily fragile if decoherence is present in an internal loop of Vaidman interferometer provided that duration of decoherence remain short with respect to overall time scales of particle motion in the Vaidman interferometer. We hope that our results, although only qualitative, can serve as a guidelines for experiments and support further investigations concerning past of quantum particles.

## Methods

For analytical calculation of pure decoherence model we utilized coherent state techniques. Numerical calculation for dissipative environment in Davies approximation we performed with Python-based toolbox QuTip^54,55^ using mesolve for Master Equation Eq. (19).
